# Sex-Related Effects of an Immune Challenge on Growth and Begging Behavior of Barn Swallow Nestlings

**DOI:** 10.1371/journal.pone.0022805

**Published:** 2011-07-27

**Authors:** Andrea Romano, Diego Rubolini, Manuela Caprioli, Giuseppe Boncoraglio, Roberto Ambrosini, Nicola Saino

**Affiliations:** 1 Dipartimento di Biologia, Università degli Studi di Milano, Milano, Italy; 2 Department of Zoology, University of Cambridge, Cambridge, United Kingdom; 3 Dipartimento di Biotecnologie e Bioscienze, Università degli Studi di Milano-Bicocca, Milano, Italy; Cajal Institute, Consejo Superior de Investigaciones Científicas, Spain

## Abstract

Parent-offspring conflicts lead the offspring to evolve reliable signals of individual quality, including parasite burden, which may allow parents to adaptively modulate investment in the progeny. Sex-related variation in offspring reproductive value, however, may entail differential investment in sons and daughters. Here, we experimentally manipulated offspring condition in the barn swallow (*Hirundo rustica*) by subjecting nestlings to an immune challenge (injection with bacterial lipopolysaccharide, LPS) that simulates a bacterial infection, and assessed the effects on growth, feather quality, expression of morphological (gape coloration) and behavioral (posture) begging displays involved in parent-offspring communication, as well as on food allocation by parents. Compared to sham-injected controls, LPS-treated chicks suffered a depression of body mass and a reduction of palate color saturation. In addition, LPS treatment resulted in lower feather quality, with an increase in the occurrence of fault bars on wing feathers. The color of beak flanges, feather growth and the intensity of postural begging were affected by LPS treatment only in females, suggesting that chicks of either sex are differently susceptible to the immune challenge. However, irrespective of the effects of LPS, parents equally allocated food among control and challenged offspring both under normal food provisioning and after a short period of food deprivation of the chicks. These results indicate that bacterial infection and the associated immune response entail different costs to offspring of either sex, but a decrease in nestling conditions does not affect parental care allocation, possibly because the barn swallow adopts a brood-survival strategy. Finally, we showed that physiological stress induced by pathogens impairs plumage quality, a previously neglected major negative impact of bacterial infection which could severely affect fitness, particularly among long-distance migratory birds.

## Introduction

Theoretical models of conflicts among family members posit that offspring are selected to obtain a larger share of parental resources than their siblings and to attract more care than would be optimal for parents to provide [1]–[3]. Because an even investment in offspring with different quality may result in a waste of reproductive effort, parents may decide to differentially invest limiting resources in relation to the reproductive value of individual offspring, in order to maximize their own fitness [2]–[6].

In altricial species from diverse taxa, offspring are entirely dependent on food provided by parents and solicit care by using morphological and behavioral ‘begging’ displays [7], [8]. Natural selection may thus have promoted the parental ability to allocate resources according to variation in offspring signals of need (e.g. hunger) and condition (general state) (see [9]). Indeed, both theoretical models and experimental studies have supported this prediction, and have suggested that multi-trait begging displays convey reliable information over offspring quality to attending parents [2], [3], [7], [10]–[13].

Given the abundance of parasites in natural environments, one important source of variation in offspring quality is parasite infection [14]–[16]. Parasites can negatively influence the physiological state of their hosts by causing disease and reducing food intake and resource assimilation [17], [18], or imposing an energy cost due to mounting an immune response which may have to be traded against competing physiological functions [19]–[21]. Negative effects of parasite infection should be more intense in young individuals that have a relatively naïve immune system [14], [16], reducing growth and survival [17], [19]. In birds, different components of offspring begging, such as postural and vocal displays and gape coloration, may reveal infection by parasites, thus potentially allowing parents to invest resources differentially according to progeny current level of infection [13], [15], [22], [23]. Begging displays may thus function as ‘honest’ signals of offspring general condition and/or reveal need of food [2], [3], [7], [10], [11], [13], [15], and several studies have demonstrated that parents respond to them by increasing food provisioning [7], [9], [24]–[27]. Importantly, the marginal fitness return of investing in offspring of different condition may vary according to contingent need of food by individual nestlings [15], [25], [27], [28].

Another crucial source of variation in offspring reproductive value is sex, as males and females may differ in susceptibility to environmental and rearing conditions [27], [29] as well as in their food demands, competitive ability and begging behavior [25], [30]. Sex-related variation in offspring fitness returns may thus promote differential parental investment in sons and daughters [4], [6]. However, despite the important role of sex in determining developmental trajectories, physiology and behavior [31], sex-specific susceptibility to parasitism of the offspring has been seldom investigated in avian species [32]–[34].

The aim of this study of barn swallow (*Hirundo rustica*) nestlings was to evaluate whether an immune challenge, simulating an infection by a bacterial pathogen, affected body mass and feather growth, as well as the expression of morphological (gape coloration) and behavioral (postural display) begging traits involved in parent-offspring communication. In addition, we evaluated variation in parental allocation strategies towards offspring differing in condition, as affected by the immune challenge, as well as by contingent need of food, as experimentally altered by a short-term food deprivation, because parental decisions and begging behavior are also expected to vary in relation to both general condition and current hunger state of the offspring (see [9]). Finally, we investigated whether male and female chicks responded differently to the immune challenge.

We simulated a bacterial infection by injecting half of the chicks of a brood with lipopolysaccharide (LPS) (LPS chicks), an endotoxin extracted from the outer membrane of Gram-negative bacteria, and the other half with a saline control solution (control chicks). LPS is commonly used to elicit an immune response in the absence of a living pathogen and causes several hormonal and behavioral alterations (the ‘sickness behavior syndrome’) in birds (e.g. [17], [18]).

We predicted that LPS depressed body mass and resulted in lower plumage quality [17], [18], [35], as gauged by feather growth and occurrence of fault bars. Fault bars are translucent bands on feathers, running perpendicular to the rachis, caused by defective development of barbules [36]. We also predicted that exposure to LPS resulted in paler coloration of chick gapes [22]. However, we had no specific predictions concerning the differential effects of LPS treatment on male and female offspring.

Finally, two days after injection, we experimentally tested the effects of the immune challenge on success in sib-sib competition between LPS and control nestlings. We measured the intensity of postural begging behavior, reflecting the degree of offspring need, and the change in body mass during feeding trials. Moreover, we counted the number of feedings provided by parents to each chick, in tests where pairs (‘dyads’) of same-sex and opposite-treatment brood-mates were set to compete for parental feedings [25], [26], [28].

## Materials and Methods

### General field procedures and sex determination

The barn swallow is a small (ca. 20 g), insectivorous migratory passerine with biparental care of the offspring. Females lay 1–3 clutches of 1–7 eggs (modal size: 5 eggs) per breeding season [37]. Nestlings hatch approximately 14 days after the onset of incubation, and fledge when they are 19–21 days old [37].

The present study was carried out between April and July 2010 at two colonies (n = 58 breeding pairs in total) located near Milan (Northern Italy). Starting from April 1^st^, nests were visited daily to record breeding events. At day 7 (day 0 = hatching of the first egg in a nest) we ringed all the chicks from broods with three or more nestlings and collected a blood sample (ca. 80 µl) for molecular sexing by PCR amplification of the sex-specific avian *CHD-1* gene following the protocol originally devised by Griffiths et al. [38], slightly modified according to Saino et al. [39]. This procedure allowed us to determine the sex of all nestlings before the day of the immune challenge.

On day 12 (mean ± SD: 11.93±0.62 days), when chicks have attained final body size and before the onset of pre-fledging mass recession [40], we intraperitoneally injected half of the male and half of the female chicks within each brood (107 males and 95 females from 47 broods) with 20 µl phosphate-buffered saline (PBS) containing 10 µg of lyophilized LPS powder, isolated from *Escherichia coli* (055:B5 - L2880 Sigma-Aldrich) (e.g. [17], [41]). Injection with LPS provokes a rapidly ensuing innate immune response (‘acute phase response’). The acute phase response triggers neuroendocrine processes, such as inhibition of the hypothalamo-pituitary-gonadal axis and activation of the hypothalamo-pituitary-adrenal axis, by release of glucocorticoids [18]. In passerines, in conjunction with these hormonal alterations, LPS causes a typical ‘sickness behavior’ by reducing activity and food intake, inducing somnolence and hypothermia, and often resulting in mass loss [17], [18], [41], [42]. Since body mass of barn swallow chicks at day 12 is ca. 20 g (20.66 g±0.12 SE in our sample of nestlings), the amount of LPS we chose to inject corresponds to ca. 0.5 µg g^−1^ body mass, a dose similar to that used in previous studies of passerines (e.g. [21], [43], [44]). The remaining nestlings were injected with the same amount of phosphate-buffered saline (PBS) to serve as controls. For example, in a brood containing two male and two female nestlings, we injected one male and one female with LPS, and one male and one female with PBS. Nestlings were assigned to the LPS or control group randomly. In case of an odd number of nestlings of either sex, the odd nestling was assigned randomly to either treatment. Overall, we injected 102 nestlings with LPS (56 males and 46 females) and 100 with PBS (51 males and 49 females).

Before LPS injection, we measured body mass to the nearest 0.1 g by an electronic balance and tarsus length with a digital caliper (to the nearest 0.01 mm). The length of all primary wing feathers (hereafter ‘primary feathers’) was also measured using a ruler (to the nearest 0.5 mm). As a proxy of wing length, we used the length of the third outermost right primary feather (‘feather length’ hereafter) [45]. Body mass, tarsus length and feather length were measured again 2 and 3 days after the injection (i.e. at day 14 and 15) to evaluate the effects of LPS on growth.

Finally, on day 17 we counted the fault bars on both wings in a subsample of 75 LPS and 73 control chicks from 35 broods. To determine which fault bars were developed after LPS treatment, we measured the length of all the feathers where fault bars were found as well the distance between each fault bar and the tip of these feathers. Since we already knew the length of all feathers before the injection of LPS or PBS, this procedure allowed us to determine the number of fault bars appearing after the immune challenge.

### Mouth coloration

On day 12 (before LPS injection), we recorded mouth coloration of 149 chicks (75 LPS and 74 control) belonging to 35 broods with a spectrometer powered by a deuterium-tungsten halogen light source (Avantes AvaSpec 2048). The reflectance (%) of the mouth was measured relative to a standard white tablet (WS-2). To prevent interference by stray light, the reflection probe was positioned inside a matte black plastic tube, cut at 45° in order to avoid specular reflection when the probe was applied to the mouth. The illuminated field was about 7 mm^2^ and every reading was obtained from an average of 15 scans. Each nestling was measured twice in two regions of the gape, corresponding to the left flange and the palate. The standard white was recalibrated before starting measurements of any next brood and the white periodically checked to verify 100% reflectance. Reflectance measures were repeated 2 and 3 days after the injection of LPS.

Color analyses were restricted to the 320–700 nm spectral window, corresponding to the typical visual range of passerines (see [46]). Spectral color composition was summarized by computing brightness, chroma and hue according to the segment classification method developed by Endler [47] and using the formulas given by Armenta et al. [46], employing *ad hoc* implemented macros for Microsoft Office Excel 2003. Brightness corresponds to the total reflectance of a given surface, chroma represents the spectral purity (saturation), while hue is the spectral location, representing the position of a spectrum in the color wheel, progressing from red to UV-A. Repeatability of the two reflectance measures of each gape region was high, with intraclass correlation coefficient [48] ranging between 0.613 (F_1,147_ = 4.152; P<0.0001) and 0.893 (F_1,148_ = 17.728; P<0.0001). Brightness, chroma and hue were thus averaged between spectra before analyses.

### Feeding trials and video recordings of begging

To test for a difference in competitive ability between LPS and control chicks, we compared the intensity of postural begging, body mass gain and number of feedings received from parents during feeding trials within pairs of same-sex and opposite-treatment siblings (i.e. either LPS male vs. control male or LPS female vs. control female) two days after experimental treatment. Pairs of same-sex chicks (dyads hereafter) were randomly chosen within each brood. The test was performed both before and after a period of food deprivation in order to analyze the behavior of nestlings and parents under different hunger conditions (normal food intake vs. hunger condition) [25], [26]. The main aim of feeding trials was to evaluate the effect of the immune challenge on competitive interactions between LPS and control nestlings. Because male and female barn swallow nestlings differ in competitive ability [25], [49] and are differently susceptible to poor rearing condition [27], including parasite loads [33], in order to experimentally control for the effect of sex we decided to establish only dyads of nestlings of the same sex.

First, the two focal nestlings were weighed, individually marked on their head with two white spots, and left in the nest for a feeding trial while temporarily removing the other chicks, that were kept in a safe and warm place. All feeding visits of the parents were videotaped with a Sony DCR-SR72E camera, placed 2–3 m from the nest in a frontal position. Recordings started in the morning at 7.30 a.m. (±30 min). At the end of the 1.5 h feeding trial, the focal nestlings were weighed again in order to record body mass gain, indicating individual food intake [25]. Afterwards, they were placed in a warm cloth bag and in a safe position for 2 h of food deprivation while their siblings were put back in the nest. Food deprivation was intended to simulate a short period of starvation, similar to what may naturally occur, for example, in case of heavy rain. The same procedure was repeated in a second feeding trial, performed after the 2 h of food deprivation. Finally, all nestlings of the brood were returned to the nest.

The number of feedings obtained by each nestling of the dyad were counted on video recordings using VLC Media Player 1.1.4 (Free Software Foundation, Inc., Boston, MA). The use of the number of feedings provided by parents to each chick during trials was intended to assess the ability of the nestlings in sib-sib competition as number of interactions won against their competitors. Moreover, because feeding rates do not account for variation in size of individual feedings, we also used body mass change during each trial as a proxy for food intake and the balance between benefits and costs of scrambling.

Furthermore, three feeding events were randomly chosen to estimate the intensity of postural begging, which was scored on a four-levels scale ranging between 0 (the nestling did not beg) and 3 (the nestling begged by moving the open wide mouth with fully stretched neck and tarsi) [26]. Feeding events were selected over the entire duration of recordings (1.5 h) for the first feeding trial while only over the first half (45 min) for the trial performed after food deprivation in order to avoid the dissipation of any effect of increased hunger level on the intensity of postural begging (see [26]). Begging scores were then averaged for each chick within a trial. All these measures were taken blindly with respect to treatment. The analyses of postural begging displays were performed both by using average begging scores for each chick within a trial as well as all the three measures for each chick within a trial.

The whole protocol was performed for 45 dyads (24 male and 21 female dyads) belonging to 39 different broods.

### Statistical analyses

The effects of immune challenge on nestling traits (body mass, feather length, color hue, chroma and brightness of both flange and palate) were analysed using linear mixed models, which included as predictors two dichotomous fixed factors for treatment (LPS or control) and sex, their interaction, and the value of the trait of interest before LPS injection as a covariate. Nest identity was included in the models as a random intercept effect. In all analyses, we also included age at LPS injection as a covariate to account for small variation in age at measurement. We first run separate analyses for trait values recorded 2 and 3 days after LPS injection, and then analysed traits recorded in both days in the same model, while including an additional fixed factor (day of measurement), identifying the data collected in either day.

The presence and number of fault bars on the wings after LPS injection were analysed in mixed models assuming a binomial and a Poisson error distribution, respectively. The presence and number of fault bars before LPS injection were also included as predictors in the relevant models to account for individual variation in the number of fault bars at the beginning of the test. Nest identity was included as a random factor in the models.

The analyses of postural begging intensity, number of feedings and body mass gain during feeding trials were carried out using repeated-measures linear mixed models where nestlings were modeled as subjects. Food deprivation (before or after) was included as a dichotomous factor identifying the repeated measurements of each subject. Sex, treatment (LPS or control) and the interaction between treatment and food deprivation were included as fixed factors. Nest and dyad were included as random intercepts, and the effect of food deprivation was allowed to vary randomly between chicks (random slope model) [50]. Because begging intensity and the outcome of sibling competition may depend both on the sex of the focal nestling and on the sex of the competitor [49], and dyads always included chicks of the same sex (see above), our experimental design did not allow to analyze the effect of the statistical interaction between sex and treatment. We therefore ran the analyses for each sex separately.

All analyses were run in R 2.8.1 (R Development Core Team 2008) with the *lmer* procedure of the *lme4* package [51]. P-values for linear mixed models were calculated by means of the likelihood ratio statistic [52]. Interaction terms were removed from the models if not significant (P>0.05). Sample size may differ slightly between different analyses because of missing data for some chicks.

### Ethics statement

When removed from their nest, nestlings were kept in a safe and warm place. At each measurement session each chick was handled only for few minutes and nests were never left without at least one nestling inside to avoid parental desertion. Blood samples were collected by slightly puncturing the brachial vein and the puncturing site was carefully disinfected. Injections of LPS and PBS were performed just below the pectoral muscle threading the tiny needle (30 G) approximately 3–4 mm inside the abdominal cavity and taking care to avoid damaging inner organs (which are easily visible through the skin at this age). No obvious negative consequences of handling nestlings were detected. Nestling mortality until fledging was very low (2 out of 102 LPS chicks and 2 out of 100 control nestlings), and unaffected by experimental treatment. During videotaping, we could not note any obvious effect derived from the presence of recording equipment on both parental and offspring behavior. The study was approved by the Ethical Committee of the Department of Biology, University of Milan, Italy.

## Results

### Body mass, feather growth and occurrence of fault bars

Before LPS treatment, no significant differences in body mass, tarsus or feather length were found between LPS and control nestlings (in all cases: χ^2^_1_≤2.339; P≥0.126).

**Figure 1:**
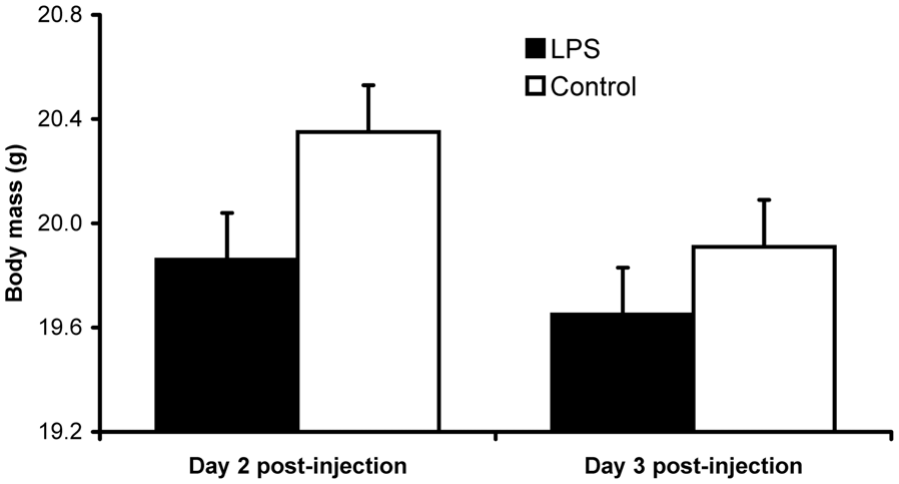
Body mass on day 2 and 3 after LPS injection. Model-estimated (see Table 1) mean body mass (+ SE) of LPS and control nestlings 2 (left) or 3 (right) days after the immune challenge.

**Figure 2:**
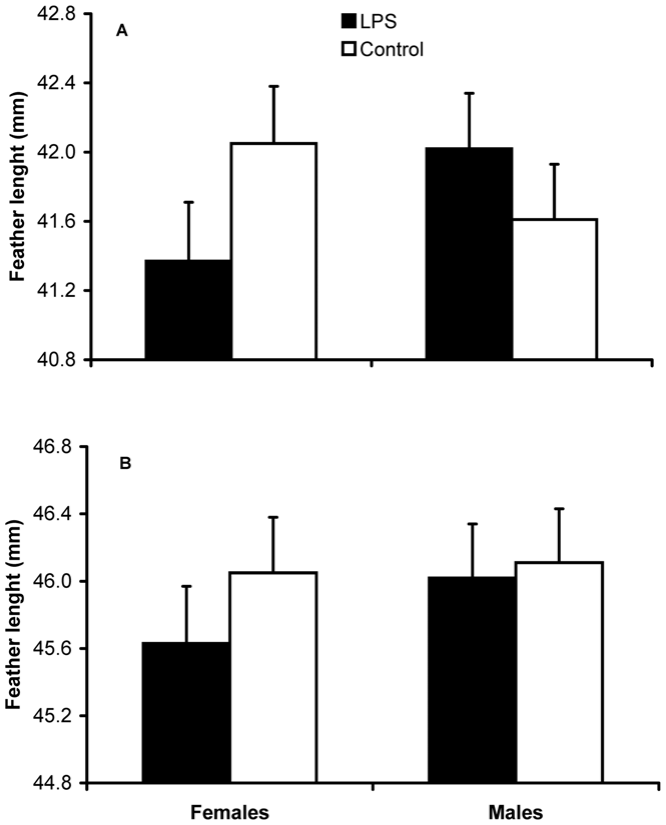
Feather length of males and females on day 2 and 3 after LPS injection. Model-estimated (see Table 1) mean third primary feather length of male and female nestlings belonging to the LPS or control group 2 (A) and 3 (B) days after the immune challenge.

On both day 2 and 3 after injection, body mass of LPS nestlings was significantly lower than that of their control siblings (Table 1; Figure 1), whereas it did not differ between sexes. On day 2 post-injection, feather length was differently affected by LPS depending on sex, with LPS females, but not males, growing shorter feathers than controls (Table 1; Figure 2), though this effect was no longer significant on day 3 post-injection (Table 1; Figure 2).

**Table 1:** Effect of LPS on body mass and feather length on day 2 and 3 post-injection.

Source of variation	Coefficient	χ^2^	df	P
**Day 2 post-injection**				
*Body mass* (n = 202)				
Treatment	−0.492 (0.087)	29.25	1	<0.0001
Sex	0.089 (0.103)	0.74	1	0.388
*Feather length* (n = 201)				
Treatment	−0.684 (0.321)	4.59	1	0.032
Sex	−0.647 (0.342)	1.90	1	0.168
Treatment×sex	−1.090 (0.444)	6.07	1	0.014
**Day 3 post-injection**				
*Body mass* (n = 193)				
Treatment	−0.251 (0.114)	4.83	1	0.028
Sex	0.053 (0.133)	0.16	1	0.691
*Feather length* (n = 193)				
Treatment	−0.244 (0.247)	0.99	1	0.320
Sex	0.216 (0.278)	0.62	1	0.430

Final models reporting the effect of treatment (LPS or control), sex and their interaction (where significant) on body mass and third primary feather length of nestlings both two and three days after the experimental manipulation. The number of nestlings in the sample is given in parentheses. See Statistical Methods for details.

Analyses run by including data from both day 2 and 3 post-injection in the same model confirmed the statistically significant effect of immune challenge on body mass (χ^2^_1_ = 15.460; P<0.0001), while the effect of treatment on feather length was non-significant (χ^2^_1_ = 2.603; P = 0.107). All the two- and three-way interaction terms between day of measurement, sex and treatment were not statistically significant (in all cases: χ^2^_1_≤2.792; P≥0.095), implying that the effects of immune challenge did not differ statistically between day 2 and 3 post-injection.

**Figure 3:**
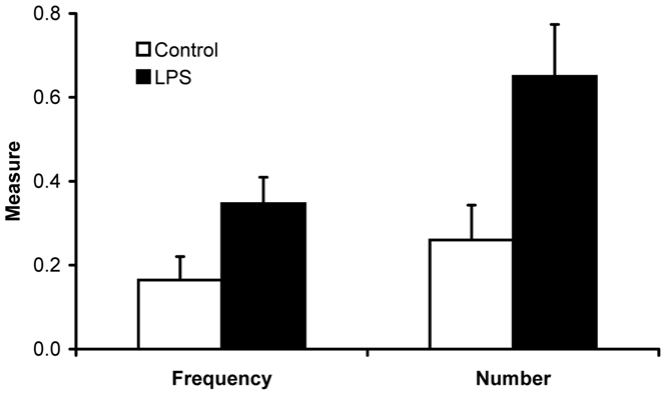
Frequency and number of fault bars on feathers in relation to LPS treatment. Proportion (+ SE) of individuals with fault bars (left) and mean number of fault bars (right) on the wings feathers of LPS (n = 75) and control (n = 73) chicks. Standard errors were calculated using the Wilson's score method incorporating continuity correction.

Before LPS injection, no differences in the presence and in the number of fault bars on wing feathers were found between LPS and control nestlings (in both cases: |z|≤0.860; P≥0.388). Conversely, post-injection, both the proportion of individuals with fault bars on wing feathers and the number of fault bars differed between treatments (Figure 3): fault bars were found on 26 out of 75 LPS nestlings but only on 12 out of 73 control chicks (z = 2.384; P = 0.017), while the absolute number of fault bars present on LPS nestlings was more than twice that on controls (Figure 3; z = 2.808; P = 0.005). No differences in presence and number of fault bars were found between male and female nestlings (in all cases: |z|≤1.401; P≥0.161). The treatment by sex interaction was not significant (in all cases: |z|≤0.170; P≥0.865) and was therefore removed from the models.

### Mouth coloration

Before LPS treatment, no significant differences in brightness, chroma and hue of the palate and the flanges were found between LPS and control nestlings (χ^2^_1_≤2.456; P≥0.117 for all color variables).

On day 2 after treatment, chroma was affected by LPS. In the palate, chroma was significantly smaller in LPS than in control nestlings (control: 0.251±0.008; LPS: 0.236±0.008; χ^2^_1_ = 6.278; P = 0.012; Table S1), while flange chroma was significantly affected by the interaction between sex and treatment (χ^2^_1_ = 6.026; P = 0.014; Table S1), with LPS reducing chroma of females (control: 0.095±0.005 SE; LPS: 0.079±0.005 SE) but not males. A sex-related variation also emerged within the control group with males having larger flange chroma than females (χ^2^_1_ = 7.950; P = 0.005; Table S1). Finally, females had larger flange hue than males (χ^2^_1_ = 6.301; P = 0.012; Table S1), while LPS and control nestlings did not differ for this variable (χ^2^_1_ = 2.169; P = 0.141).

On day 3 post-injection, no effects of treatment (in all cases: χ^2^_1_≤2.231; P≥0.135) or sex (in all cases: χ^2^_1_≤3.470; P≥0.062) were found on any color variable (Table 2).

**Table 2:** Effect of LPS on intensity of postural begging, number of feedings received and body mass gain during feeding trials.

Source of variation	Coefficient	χ2	df	P
**All nestlings**				
*Postural begging* (n = 90)				
Sex	−0.092 (0.153)	0.362	1	0.548
Treatment	0.236 (0.107)	4.570	1	0.033
Food deprivation	0.184 (0.087)	4.436	1	0.035
*Number of feedings* (n = 90)				
Sex	−0.256 (1.332)	0.054	1	0.816
Treatment	0.050 (0.590)	0.007	1	0.935
Food deprivation	4.965 (0.633)	47.995	1	<0.0001
*Body mass variation* (n = 90)				
Sex	0.097 (0.119)	0.676	1	0.411
Treatment	0.008 (0.047)	0.029	1	0.865
Food deprivation	0.537 (0.064)	52.694	1	<0.0001
**Male nestlings**				
*Postural begging* (n = 48)				
Treatment	0.123 (0.142)	0.765	1	0.382
Food deprivation	0.286 (0.120)	5.367	1	0.021
*Number of feedings* (n = 48)				
Treatment	−0.376 (0.925)	0.172	1	0.678
Food deprivation	5.495 (0.977)	24.632	1	<0.0001
*Body mass variation* (n = 48)				
Treatment	0.008 (0.075)	0.011	1	0.915
Food deprivation	0.494 (0.090)	29.162	1	<0.0001
**Female nestlings**				
*Postural begging* (n = 42)				
Treatment	0.366 (0.161)	4.939	1	0.026
Food deprivation	0.082 (0.122)	0.466	1	0.495
*Number of feedings* (n = 42)				
Treatment	0.937 (0.619)	1.959	1	0.162
Food deprivation	4.334 (0.757)	29.168	1	<0.0001
*Body mass variation* (n = 42)				
Treatment	0.010 (0.053)	0.037	1	0.847
Food deprivation	0.059 (0.090)	29.733	1	<0.0001

Final models reporting the effect of treatment and food deprivation on postural begging, feedings received by individual offspring and body mass variation of nestlings in a repeated-measures linear mixed model. The analyses were performed for the entire set of nestlings, and for each sex separately. The number of nestlings in the sample is given in parentheses. See Statistical Methods for details.

Analyses including data from both day 2 and 3 after LPS injection (see Statistical analyses) confirmed the significant main effect of the immune challenge on palate chroma (χ^2^_1_ = 4.951; P = 0.026) as well as of the interaction between sex and LPS treatment on flange chroma (χ^2^_1_ = 4.556; P = 0.033). These models also confirmed the sex-related variation in the flange chroma (χ^2^_1_ = 4.746; P = 0.029) (see above). In addition, the other two- and three-way interaction terms between day of measurement, sex and/or LPS were not statistically significant (in all cases: χ^2^_1_≤3.116; P≥0.078), implying that the effects of LPS treatment were not significantly different between day 2 and 3.

### Feeding trials

Repeated-measures mixed models showed that average begging intensity was higher among LPS compared to control chicks (Table 2), whereas there was no effect of treatment on food allocation and body mass gain during feeding trials (Table 2). Average begging intensity, the number of feedings received per capita and body mass gain during feeding trials significantly increased after food deprivation (Table 2).

**Figure 4:**
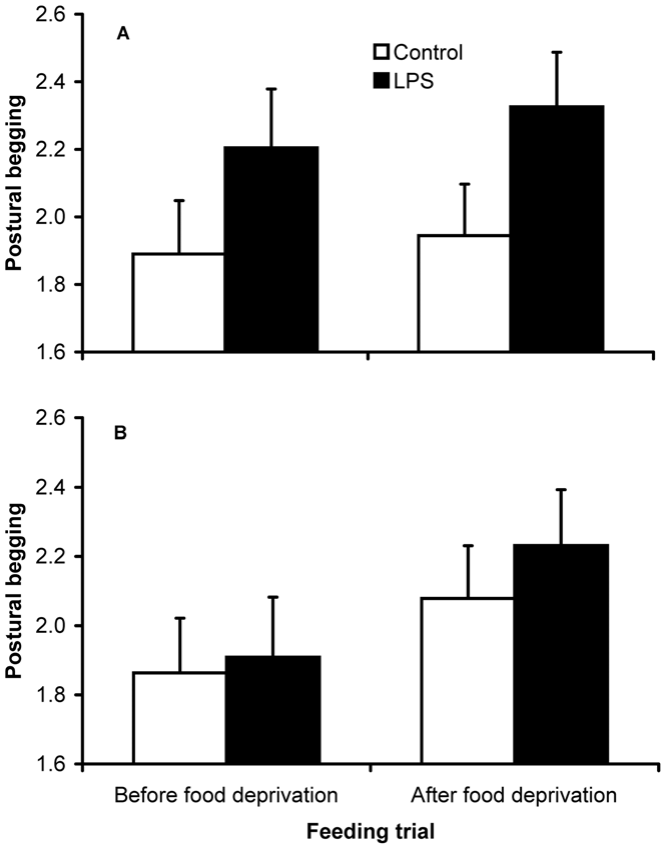
Begging intensity before and after food deprivation in relation to LPS treatment. Model-estimated (see Table 2) mean intensity (+ SE) of postural begging display in 21 dyads of female (A) and 24 dyads of male (B) nestlings, before and after a period of 2 hours of food deprivation.

Separate analyses for each sex revealed no significant effect of LPS treatment on average begging intensity, food allocation and body mass gain in males, though all these variables were strongly affected by food deprivation (Figure 4; Table 2). Conversely, LPS females begged more intensely than their control sisters (Figure 4) but did not receive more food from parents nor did they gain more mass (Table 2). Interestingly, among female nestlings, both the number of feedings obtained and the body mass gain significantly increased after food deprivation, while begging intensity increased slightly but not significantly (Table 2).

Qualitatively similar results were obtained by including in the model all three available scores of begging intensity for each chick rather than using individual averages computed within trial (details not shown for brevity).

## Discussion

In this experiment on barn swallow nestlings, we subjected chicks to an immune challenge with LPS, and assessed its effects on growth, feather quality and parent-offspring communication. Infection by Gram-negative bacteria can elicit an immune response and cause an ‘acute phase response’, entailing physiological and behavioral alterations [18], [42]. As predicted, the immune challenge negatively affected several morphological traits such as body mass, palate color and feather quality as reflected by the occurrence of fault bars on wing feathers. The effect of LPS on wing feathers growth and beak flange coloration differed between males and females, as suggested by the significant sex by treatment interaction. Moreover, the LPS injection determined an increase in begging intensity of females but not of males. Albeit a direct comparison between sexes was prevented by our experimental design (see Methods), the difference in begging intensity between LPS and control females was three times larger than that observed among males, thus suggesting that also in this case the effect of the immune challenge may be sex-specific. However, LPS injection did not affect parental feeding effort both under a normal food provisioning regime and after a short period of food deprivation. Below we discuss the main findings.

### Effects on morphological traits and feather quality

Loss of body mass and reduced feather development and quality (as gauged by the slower growth of primary feathers in females and by a larger occurrence of fault bars) following LPS challenge may have been caused by a smaller food intake. In passerine birds the acute phase response is associated with an increase in resting and somnolence as well as a reduction of behaviors associated with motility, like scrambling for food and sib-sib competitive interactions (the ‘sickness behavior syndrome’) [17], [18], [41], [42]. These behavioral changes are typically short-lasting and individuals recover within 24 hours [18], [42]. Nevertheless, other effects such as mass loss and growth reduction may persist for longer [17], [18], [41].

A decrease in body mass may also have been caused by a reduction in parental feeding soon after the immune challenge, either because LPS chicks may appear of reduced reproductive value to parents, or because their nest-mates prevailed in sib-sib interactions for access to food. Although we admittedly could not discriminate between these interpretations, we favor the idea that reduced access to food was mainly due to reduced motility. This is the case because previous studies on barn swallows suggested that both parents and older/larger chicks seem to enhance access to food by smaller nestlings, as expected in a species adopting a brood survival strategy [25], [26], [28], [49].

Our findings also highlighted a possible trade-off between growth and immunity [53], [54]. Functioning of the immune system is costly, and energy trade-offs among competing functions may be more intense in rapidly growing young individuals [55], [56]. LPS nestlings may thus have used most of their available energy to mount an immune response, thus suffering a reduction in their body and feather growth, as observed in other bird species [19], [20], [35].

A novel finding is that the immune challenge increased the occurrence of fault bars, that are commonly considered as evidence of low feather quality [36]. Indeed, their presence is associated with higher risk of breakage [57], [58] and with other major feather damages [59], that may result in a considerable reduction in aerodynamics and flight performance [60]. Feather damage may thus have consequences for aerial foraging, predator escape behavior, and migration performance [58] in this long-distance migratory bird. This finding corroborates previous evidence of impaired plumage growth consequent to an immune stimulation in molting adult house sparrows (*Passer domesticus*) [35]. Though the proximate mechanisms remain unknown, the negative effects of LPS injection on feather quality and growth may be mediated by corticosterone. Exposure to LPS is known to raise circulating corticosterone levels [18] and high corticosterone, in turn, reduces the number of barbules and affects their reciprocal distance [61], [62], resulting in increased frequency of fault bars. Moreover, because nutritional stress is a main cause of fault bars [63], [64], the effects of a corticosterone-mediated pathway may have been amplified by decreased food intake, as nutritional stress is known to significantly increase circulating corticosterone [63], [64].

### Effects on parent-offspring communication and sibling competition

We found that saturation of gape color, which is a main component of offspring begging signals, was depressed by LPS. The coloration of the soft tissues of the gape is partly dependent on the presence of dietary carotenoids [43], [65], and a reduced chroma of the palate (both sexes) and flanges (females only) in LPS nestlings may therefore reflect a reduction in carotenoid assimilation. Furthermore, activation of an immune response by the LPS challenge could have increased the mobilization of carotenoids, which have important immuno-modulatory functions, from gape tissues because of a trade-off in allocation of these limiting dietary components to the competing functions of gape coloration or immunity [43], [66], [67]. This interpretation is consistent with the results of previous studies showing a negative effect of an immune challenge on gape pigmentation of barn swallow nestlings [13], [22].

Female, but not male, LPS nestlings showed significantly higher postural begging scores than control nestlings, irrespective of food deprivation. These intense begging solicitations of LPS females were probably caused by their impaired condition, as reflected by negative effects of LPS on other traits. Similar results were found in a previous study of begging in nestling great tits (*Parus major*) experimentally infested by ectoparasites, with infested broods increasing their begging rate [15]. Interestingly, control females did not significantly increase their solicitation displays after food deprivation. Females have been shown to be less susceptible to food shortage than males [25], and for this reason have been hypothesized not to escalate their begging output when needy siblings are also present [25]. Thus, this finding might corroborate previous finding of state sensitivity and favoritism towards needy kin in this species [25], [26], [28], [49]. On the other hand, control males begged as vigorously as their LPS male siblings, probably because male barn swallow chicks compete for food more harshly than female chicks [25], [49].

Parents did not respond to increased postural begging of LPS females by preferentially feeding them, as assessed both by parental feeding rates and mass gain during feeding trials, possibly because they relied on other components of begging, like gape coloration, besides postural displays. In fact, LPS females had less saturated palate and flange coloration than controls of the same sex. Duller gape coloration may thus have compensated for the effect of increased postural begging on parental decisions. However, this interpretation does not apply to LPS males which had less saturated palate coloration but did not beg more intensely than controls.

An alternative interpretation is that the negative effects of the immune challenge on condition may be transient [17], [18], [41], [42], [44] and not strong enough to justify a parental favoritism towards specific nestlings. In fact, we recorded just two episodes of mortality out of 102 LPS nestlings, which corresponds to baseline mortality among barn swallow nestlings under natural conditions [37]. This interpretation implies that parents can assess whether deterioration in offspring condition is ephemeral thus avoiding sacrifice of chicks whose state can easily be improved by a relatively small additional investment, and would be generally consistent with the observation that the barn swallow is a species adopting a ‘brood-survival’ strategy [37], [40].

In conclusion, we showed that exposure to a Gram-negative bacterial endotoxin has diverse, detrimental effects on growth and begging behavior of barn swallow chicks. These effects were more evident among female chicks, disclosing an important role of bacterial infection during early life in determining sex-related differential growth and condition. Our findings further suggest that parasite infection during critical phases of feather growth, like early development or moulting, might be regarded as a cause of variation in feather quality.

## Supporting Information

Table S1 **Effects of LPS challenge on hue, chroma and brightness of gape and flanges.** Table shows final mixed models reporting the effects of treatment, sex and their interaction (where statistically significant) on hue, chroma and brightness of palate and flanges of barn swallows nestlings at both day 2 and 3 after LPS injection.
(PDF)
