# Supplementary material for: Sex-Related Effects of an Immune Challenge on Growth and Begging Behavior of Barn Swallow Nestlings
Source: PLoS One. 2011 Jul 27;6(7):e22805. doi: 10.1371/journal.pone.0022805 (PMC3144944; doi:10.1371/journal.pone.0022805)
Supplement: Table S1 — Effects of LPS challenge on hue, chroma and brightness of gape and flanges. Table shows final mixed models reporting the effects of treatment, sex and their interaction (where statistically significant) on hue, chroma and brightness of palate and flanges of barn swallows nestlings at both day 2 and 3 after LPS injection. (PDF) [file pone.0022805.s001.pdf]

Table S1. Effects of LPS challenge on hue, chroma and brightness of gape and flanges. Table shows final mixed models reporting the effects of treatment, sex and their interaction (where statistically significant) on hue, chroma and brightness of palate and flanges of barn swallows nestlings at both day 2 and 3 after LPS injection.

Source of variation	Coefficient	χ^2	df	P
**Hue palate**				
Day 2 post-injection (n = 148)				
Treatment	-0.033 (0.020)	2.660	1	0.103
Sex	-0.036 (0.023)	2.389	1	0.122
Day 3 post-injection (n = 146)				
Treatment	-0.010 (0.018)	0.327	1	0.568
Sex	0.038 (0.021)	3.359	1	0.067
**Hue flange**				
Day 2 post-injection (n = 148)				
Treatment	-0.052 (0.036)	2.169	1	0.141
Sex	-0.101 (0.041)	6.301	1	0.012
Day 3 post-injection (n = 146)				
Treatment	-0.053 (0.038)	2.008	1	0.156
Sex	-0.010 (0.042)	0.054	1	0.815
**Chroma palate**				
Day 2 post-injection (n = 148)				
Treatment	-0.015 (0.006)	6.278	1	0.012
Sex	0.002 (0.007)	0.116	1	0.733
Day 3 post-injection (n = 146)				
Treatment	-0.008 (0.005)	2.231	1	0.135
Sex	0.011 (0.006)	3.470	1	0.062
**Chroma flange**				
Day 2 post-injection (n = 148)				
Treatment	-0.016 (0.007)	5.591	1	0.018
Sex	-0.020 (0.065)	7.950	1	0.005
Treatment × sex	0.0023 (0.009)	6.026	1	0.014
Day 3 post-injection (n = 146)				
Treatment	0.004 (0.006)	0.430	1	0.512
Sex	-0.009 (0.006)	2.029	1	0.154
**Brightness palate**				
Day 2 post-injection (n = 148)				
Treatment	-0.011 (0.008)	2.11	1	0.147
Sex	-0.006 (0.009)	0.442	1	0.506
Day 3 post-injection (n = 146)				
Treatment	0.006 (0.007)	0.929	1	0.335
Sex	0.002 (0.008)	0.003	1	0.855
**Brightness flange**				
Day 2 post-injection (n = 148)				
Treatment	0.000 (0.001)	2.18	1	0.996
Sex	0.013 (0.015)	0.74	1	0.391
Day 3 post-injection (n = 146)				
Treatment	-0.008 (0.016)	0.290	1	0.590
Sex	0.031 (0.029)	2.983	1	0.084
